# Supplementary material for: Placental secretome characterization identifies candidates for pregnancy complications
Source: Commun Biol. 2021 Jun 8;4:701. doi: 10.1038/s42003-021-02214-x (PMC8187406; doi:10.1038/s42003-021-02214-x)
Supplement: Supplementary file 2 — Supplementary Information [file 42003_2021_2214_MOESM2_ESM.pdf]

Supplementary Materials

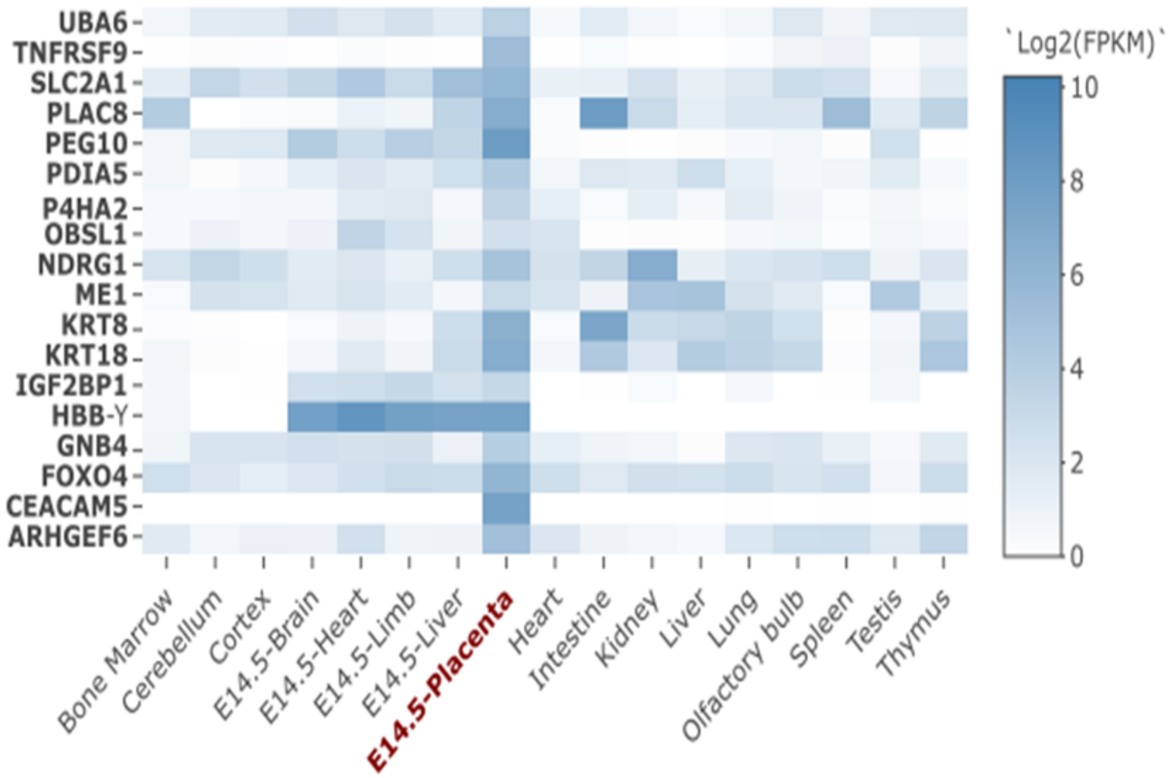

Fig. S1. Tissue enrichment analysis of the non-secreted proteins detected in the sorted mouse placental endocrine cells (Jz +*Tpbpa* sorted cells). 18 non-secreted proteins were enriched (>10 fold) in the placenta compared to other tissues in mouse using TissueEnrich.

a

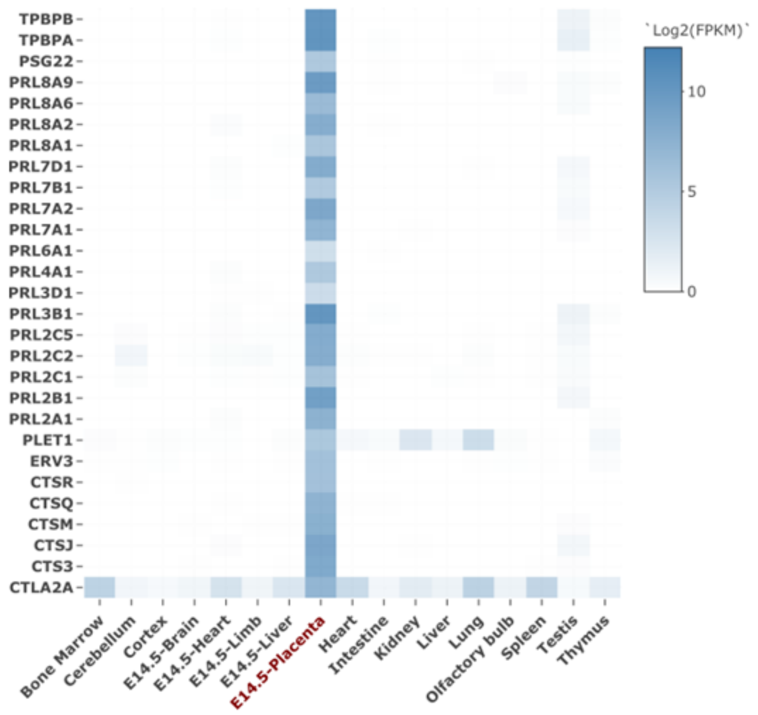

b

Biological Process

#Term ID	Term description	# obs.	FDR
GO:0010469	Regulation of signaling receptor activity	14	6.45E-13
GO:0065009	Regulation of molecular function	15	0.00023
GO:0048583	Regulation of response to stimulus	15	0.00071

Molecular Function

#Term ID	Term description	# obs.	FDR
GO:0005179	Hormone activity	14	1.39E-22
GO:0098772	Molecular function regulator	15	1.68E-09
GO:0008234	Cysteine-type peptidase activity	5	1.48E-05
GO:0004197	Cysteine-type endopeptidase activity	3	0.00073
GO:0005515	Protein binding	16	0.0107

Protein Domains

#Term ID	Term description	# obs.	FDR
MMU-8939242	RUNX1 regulates transcription of genes involved in differentiation of keratinocytes	4	1.07E-07
MMU-1679131	Trafficking and processing of endosomal TLR	4	2.83E-07
MMU-1442490	Collagen degradation	4	1.04E-05
MMU-2022090	Assembly of collagen fibrils and other multimeric structures	4	1.04E-05
MMU-2132295	MHC class II antigen presentation	21	4.99E-08

Fig. S2. Analysis of the 31 secreted proteins in the placental secretome map reported to be expressed by the mouse but not human placenta. a The expression of the 28 out of the 31 mouse-specific secreted placental proteins that were enriched (>10-fold) in the placenta compared to other tissues using TissueEnrich. **b** Gene ontology (GO) analysis for the 31 mouse-specific secreted placental proteins using STRING V.11.

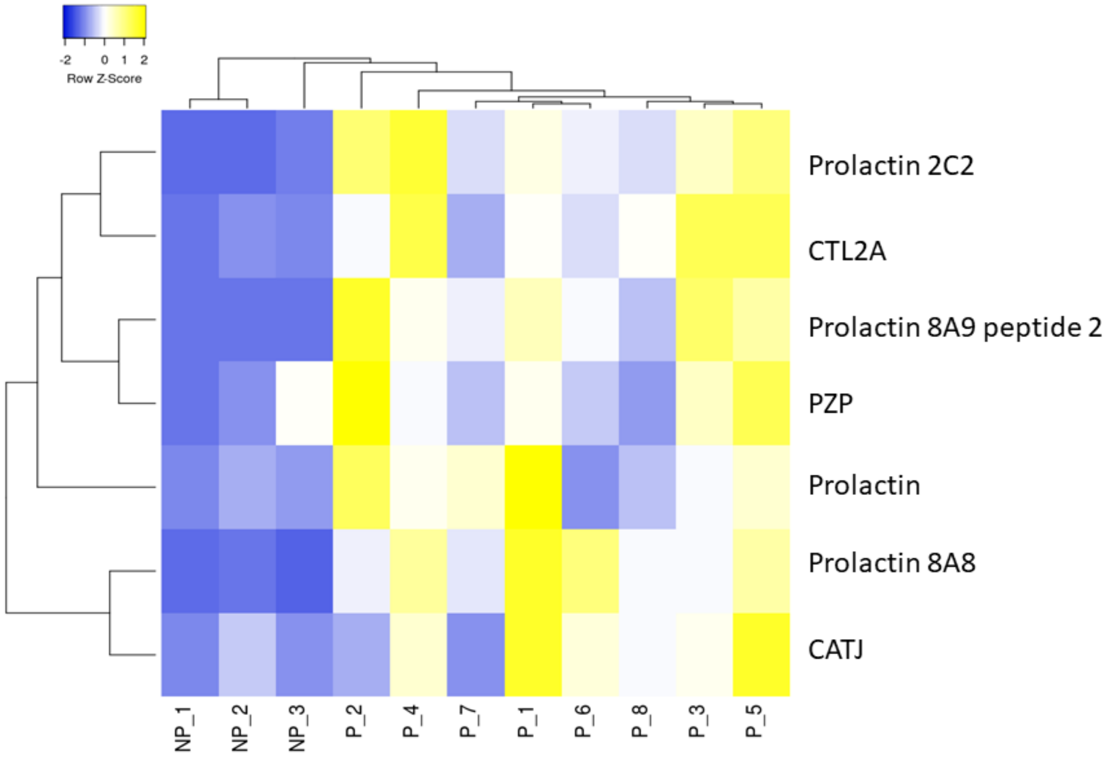

Fig. S3. Heat map showing the relative abundance of the 7 mouse placenta specific proteins in mouse non-pregnant (NP, n=3) and pregnant (day 16 of pregnancy, P, n=8) plasma. Heat map generated using Heatmapper.

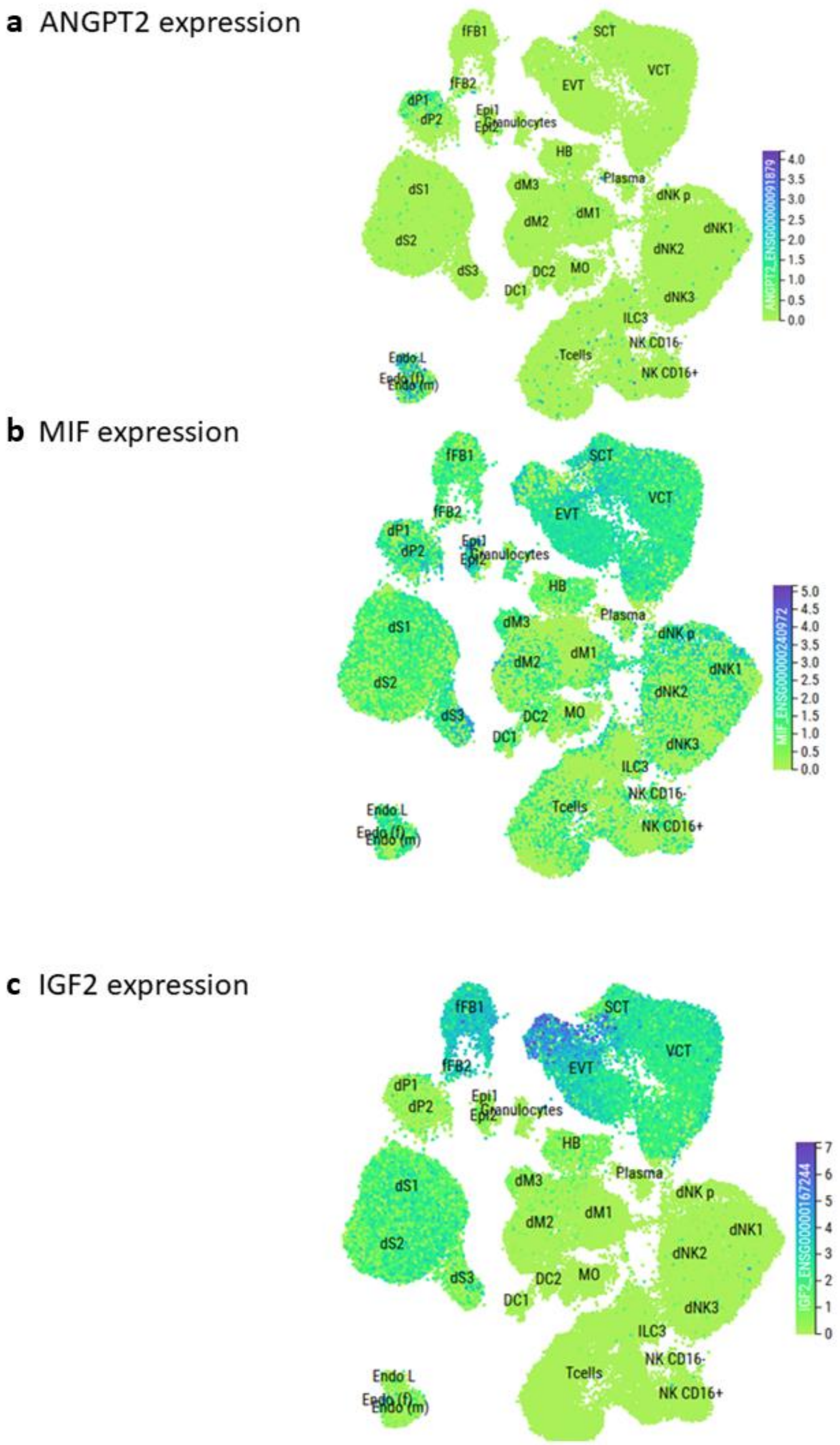

Fig. S4. Expression of placenta proteins at the maternal-fetal interface in early human pregnancy. **a** ANGPT2, **b** MIF and **c** IGF2 expression at the maternal–fetal interface of early human pregnancy via the CellxGene tool (https://maternal-fetal-interface.cellgeni.sanger.ac.uk/).

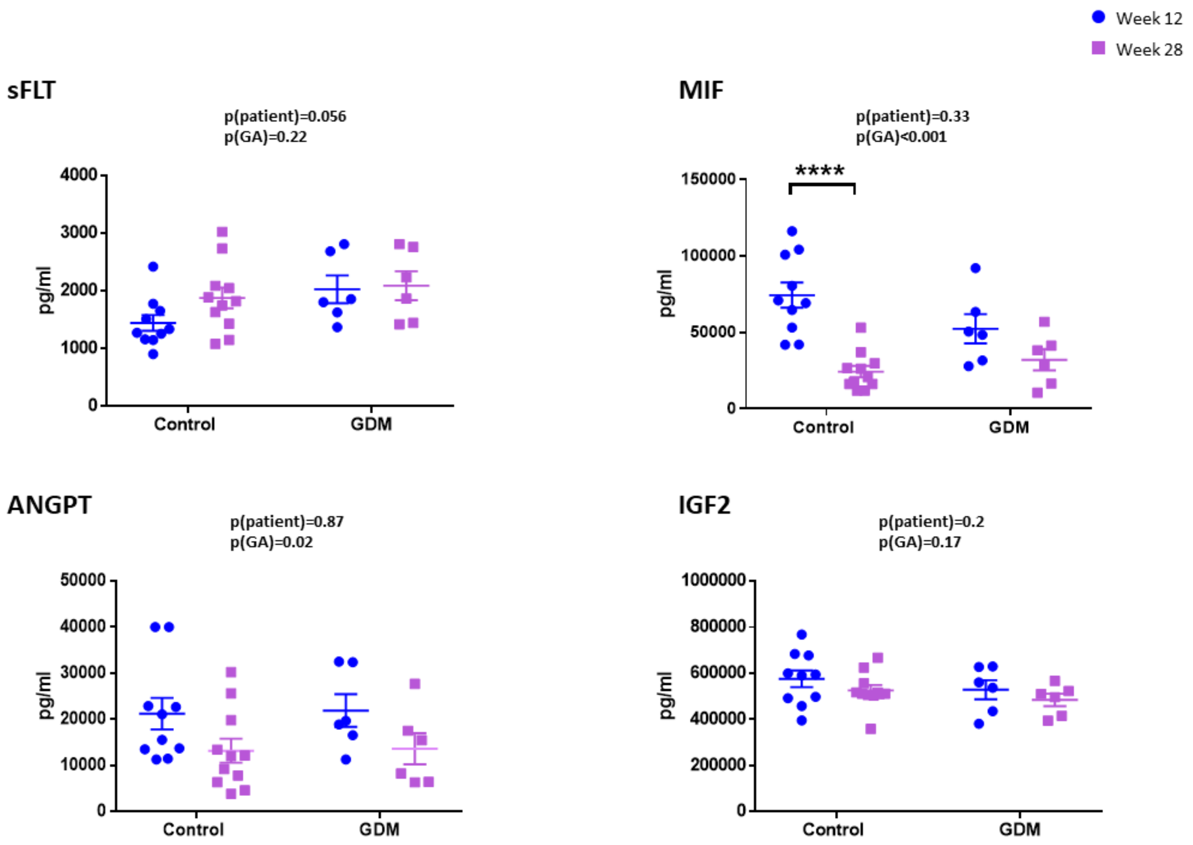

Fig. S5. Concentrations of placenta proteins in human pregnancy samples. sFLT1, MIF, ANGPT2 and IGF2 concentrations in healthy and GDM pregnancies at week 12 and 28 of gestation. Data are from n=6-10 pregnancies and shown as mean ± SEM. Asterisks denote statistical significance between week of pregnancy, using Two-way ANOVA and ****P<0.001 (there was no significant difference between control and GDM). GA: gestational age.

Species	RNA-Seq Annotation	N
**Mouse**	GSE79121	n=1 pooled litter (day 20)
	GSE11224	n=2-3 pooled litters (day 8.5 – day 20)
	GSE11224	n=2-3 pooled litters (day 8.5 - day 20)
**Human**	GSE9984	n=4 first trimester<br>n=4 second trimester<br>n=4 term placentas
	GSE28551	n=16 first trimester<br>n=21 third trimester
	GSE10588<br>GSE25906<br>GSE4707<br>GSE30186<br>GSE24129<br>GSE44711	n=26 (term placenta)<br>n=37 (term placenta)<br>n=4 (term placenta)<br>n=6 (term placenta)<br>n=8 (term placenta)<br>n=8 (term placenta)

Table S1. RNA expression database for mouse and human placenta.

DAMP proteins in 319 proteins define the "placental secretome"	Detected in mouse plasma in this study	Detected in non-pregnant mouse plasma by others	Detected in the sorted cells
PGS1_MOUSE	No	No	No
CALR_MOUSE	No	Yes[1]	Yes
PGS2_MOUSE	Yes	Yes[1,2]	No
FINC_MOUSE	Yes	Yes[1,2]	No
GPC1_MOUSE	No	Yes[1]	No
H2B1C_MOUSE	No	No	Yes
H2B1F_MOUSE	No	Yes[1]	Yes
H2B1K_MOUSE	No	No	Yes
HMGB1_MOUSE	Yes	Yes[1]	No
HS90A_MOUSE	No	Yes[1]	Yes
HS90B_MOUSE	No	Yes[1,2]	Yes
ENPL_MOUSE	No	Yes[1]	Yes
HSP74_MOUSE	Yes	Yes[1]	Yes
BIP_MOUSE	No	Yes[2]	No
HSP7C_MOUSE	No	Yes[1,2]	Yes
HS105_MOUSE	No	No	Yes
IL1R2_MOUSE	No	Yes[1]	No
PPIA_MOUSE	Yes	Yes[1]	Yes
S10AA_MOUSE	No	No	Yes
S10AB_MOUSE	Yes	Yes[1,2]	Yes
S10A6_MOUSE	Yes	Yes[1]	Yes
S10A9_MOUSE	Yes	Yes[1,2]	No
SCRB2_MOUSE	No	Yes[1]	No
TENA_MOUSE	No	Yes[1,2]	No

Table S2. Table of putative DAMP (damage-associated molecular pattern) proteins in the 319 proteins that define the "placental secretome". Potential DAMPs were based on [3].

Complication	Diagnosis of complication	Data sets available	RNA or Protein	Number of samples per sample type	Reference
**PE**	Hypertension (systolic blood pressure ≥160 mmHg and/or diastolic blood pressure ≥ 110 mmHg) after week 20 with at least one of the following symptoms: thrombocytopenia, impaired liver function, new development of renal insufficiency, proteinuria >5 g in 24 hours, new-onset cerebral or visual disturbances	4	RNA<br>RNA<br>RNA<br><br><br><br>RNA	n=8 (term placenta)<br>n=12 (term placenta)<br>n=4 (syncytiotrophoblasts*)<br>n=3 (invasive cytotrophoblasts*)<br>n=4 (endovascular cytotrophoblasts*)<br>n=77 (7 microarray studies combined^)	[4]<br>[5]<br>[6]<br><br><br><br>[7]
**GDM**	Glucose intolerance determined using an oral glucose tolerance test (75g) at 24-28 weeks of gestation and revealed by either a fasting	6	RNA<br>RNA<br>RNA<br>RNA<br>RNA<br>Protein	n=8 (term placenta)<br>n=12 (term placenta)<br>n=19 (term placenta)<br>n=7 (term placenta)<br>n=4 (term placenta)	[4]<br>[5]<br>[8]<br>[9]<br>[10]<br>[11]

	venous plasma glucose level of >5.1 mmol/l, and/or >10 mmol/l and > 8.5 mmol/l at 1h and 2h, respectively			n=135 (first trimester serum)	
**SGA**	Birth weight < 10th centile	2	RNA<br>RNA	n=8 (term placenta)<br>n=12 (term placenta)	[4]<br>[5]
**LGA**	Birth weight > 90th centile	2	RNA<br>RNA	n=8 (term placenta)<br>n=12 (term placenta)	[4]<br>[5]
**IUGR**	Abnormal Doppler waveforms in the umbilical or middle cerebral artery (both <10th centile) and/or fetal weight and/or abdominal circumference (both <10th centile) and/or head circumference (>10th centile)	3	RNA<br>RNA<br>RNA	n=5 (term placenta)<br>n=12 (third trimester, placental RNA from blood<br>n=5 (term placenta)	[12]<br>[13]<br>[4]

Table S3. Compilation of publicly available RNA and protein expression datasets for the human placenta from complicated pregnancies. * From cell populations isolated by laser capture microdissection on human placenta from pre-term pregnancies. ^ Compilation of 7 microarray datasets for the human placenta of PE pregnancies. Note there were inconsistencies between studies in the diagnosis and inclusion criteria for these 7 microarray datasets for PE.

References:

1 Yang, Y. R. *et al.* Plasma proteomic profiling of young and old mice reveals cadherin-13 prevents age-related bone loss. *Aging (Albany NY)* **12**, 8652-8668, doi:10.18632/aging.103184 (2020).

2 Michaud, S. A. *et al.* Molecular phenotyping of laboratory mouse strains using 500 multiple reaction monitoring mass spectrometry plasma assays. *Commun Biol* **1**, 78, doi:10.1038/s42003-018-0087-6 (2018).

3 Roh, J. S. & Sohn, D. H. Damage-Associated Molecular Patterns in Inflammatory Diseases. *Immune Netw* **18**, e27, doi:10.4110/in.2018.18.e27 (2018).

4 Sober, S. *et al.* Extensive shift in placental transcriptome profile in preeclampsia and placental origin of adverse pregnancy outcomes. *Sci Rep* **5**, 13336, doi:10.1038/srep13336 (2015).

5 Uuskula, L. *et al.* Mid-gestational gene expression profile in placenta and link to pregnancy complications. *PLoS One* **7**, e49248, doi:10.1371/journal.pone.0049248 (2012).

6 Gormley, M. *et al.* Preeclampsia: novel insights from global RNA profiling of trophoblast subpopulations. *American journal of obstetrics and gynecology* **217**, 200 e201-200 e217, doi:10.1016/j.ajog.2017.03.017 (2017).

7 Leavey, K., Bainbridge, S. A. & Cox, B. J. Large scale aggregate microarray analysis reveals three distinct molecular subclasses of human preeclampsia. *PLoS One* **10**, e0116508, doi:10.1371/journal.pone.0116508 (2015).

8 Enquobahrie, D. A., Williams, M. A., Qiu, C., Meller, M. & Sorensen, T. K. Global placental gene expression in gestational diabetes mellitus. *Am J Obstet Gynecol* **200**, 206 e201-213, doi:10.1016/j.ajog.2008.08.022 (2009).

9 Radaelli, T., Varastehpour, A., Catalano, P. & Hauguel-de Mouzon, S. Gestational diabetes induces placental genes for chronic stress and inflammatory pathways. *Diabetes* **52**, 2951-2958, doi:10.2337/diabetes.52.12.2951 (2003).

10 Bari, M. F., Ngo, S., Bastie, C. C., Sheppard, A. M. & Vatish, M. Gestational diabetic transcriptomic profiling of microdissected human trophoblast. *J Endocrinol* **229**, 47-59, doi:10.1530/JOE-15-0424 (2016).

11 Ravnsborg, T. *et al.* First-trimester proteomic profiling identifies novel predictors of gestational diabetes mellitus. *PLoS One* **14**, e0214457, doi:10.1371/journal.pone.0214457 (2019).

12 Majewska, M. *et al.* Placenta Transcriptome Profiling in Intrauterine Growth Restriction (IUGR). *Int J Mol Sci* **20**, doi:10.3390/ijms20061510 (2019).

13 Whitehead, C. L. *et al.* Identifying late-onset fetal growth restriction by measuring circulating placental RNA in the maternal blood at 28 weeks' gestation. *Am J Obstet Gynecol* **214**, 521 e521-521 e528, doi:10.1016/j.ajog.2016.01.191 (2016).
