# Supplementary material for: Placental secretome characterization identifies candidates for pregnancy complications
Source: Commun Biol. 2021 Jun 8;4:701. doi: 10.1038/s42003-021-02214-x (PMC8187406; doi:10.1038/s42003-021-02214-x)
Supplement: Supplementary file 3 — Description of Supplementary Files [file 42003_2021_2214_MOESM3_ESM.pdf]

Description of Additional Supplementary Files

File name: Supplementary Data 1

Description: *Gene ontology and protein domain analysis for non-secreted proteins detected in sorted mouse placental endocrine cells (Jz +Tpbpa sorted cells).* **a** Gene ontology performed by Panther analysis and **b** Gene ontology performed using Reactome pathways performed by STRING V.11.

File Name: Supplementary Data 2

Description: *Gene ontology (GO) analysis for the 319 secreted proteins detected in placental secretome map.* **a** Gene ontology: Molecular function, **b** Gene ontology: Protein domain, c Gene ontology: Biological process. Analyses performed using STRING V.11.

File Name: Supplementary Data 3

Description: Peptide peak area ratios for the 46 of the proteins in the placental secretome map detectable in mouse non-pregnant (NP) and pregnant (P; day 16) plasma.

File Name: Supplementary Data 4

Description: Peptide peak area ratios for the 7 mouse placenta specific proteins in mouse non-pregnant (NP) and pregnant (P; day 16) plasma.

File Name: Supplementary Data 5

Description: *Gene ontology and protein domain analysis for secreted placental proteins uniquely altered in gestational diabetes mellitus.* Gene ontology performed using Reactome pathways performed by STRING V.11.

File Name: Supplementary Data 6

Description: *Gene ontology and protein domain analysis for secreted placental proteins uniquely altered in preeclampsia.* Gene ontology performed using Reactome pathways performed by STRING V.11.

File name: Supplementary Data 7

Description: *Gene ontology and protein domain analysis for secreted placental proteins uniquely altered in intrauterine growth restriction.* Gene ontology performed using Reactome pathways performed by STRING V.11.

File name: Supplementary Data 8

Description: The 33 transcription factors (TFs) regulating the expression of genes encoding proteins in the placental secretome map with indication of the target genes differentially expressed in the placenta in pregnancy complications.
