# Supplementary material for: Placental secretome characterization identifies candidates for pregnancy complications
Source: Commun Biol. 2021 Jun 8;4:701. doi: 10.1038/s42003-021-02214-x (PMC8187406; doi:10.1038/s42003-021-02214-x)
Supplement: Supplementary file 12 — Reporting Summary [file 42003_2021_2214_MOESM12_ESM.pdf]

nature research

Corresponding author(s): Amanda N. Sferruzzi-Perri

Last updated by author(s): May 8, 2021

Reporting Summary

Nature Research wishes to improve the reproducibility of the work that we publish. This form provides structure for consistency and transparency in reporting. For further information on Nature Research policies, see our Editorial Policies and the Editorial Policy Checklist.

Statistics

For all statistical analyses, confirm that the following items are present in the figure legend, table legend, main text, or Methods section.

n/a	Confirmed	
☐	☒	The exact sample size (n) for each experimental group/condition, given as a discrete number and unit of measurement
☐	☒	A statement on whether measurements were taken from distinct samples or whether the same sample was measured repeatedly
☐	☒	The statistical test(s) used AND whether they are one- or two-sided<br>*Only common tests should be described solely by name; describe more complex techniques in the Methods section.*
☒	☐	A description of all covariates tested
☐	☒	A description of any assumptions or corrections, such as tests of normality and adjustment for multiple comparisons
☐	☒	A full description of the statistical parameters including central tendency (e.g. means) or other basic estimates (e.g. regression coefficient) AND variation (e.g. standard deviation) or associated estimates of uncertainty (e.g. confidence intervals)
☐	☒	For null hypothesis testing, the test statistic (e.g. F, t, r) with confidence intervals, effect sizes, degrees of freedom and P value noted<br>*Give P values as exact values whenever suitable.*
☒	☐	For Bayesian analysis, information on the choice of priors and Markov chain Monte Carlo settings
☒	☐	For hierarchical and complex designs, identification of the appropriate level for tests and full reporting of outcomes
☒	☐	Estimates of effect sizes (e.g. Cohen's d, Pearson's r), indicating how they were calculated

Our web collection on statistics for biologists contains articles on many of the points above.

Software and code

Policy information about availability of computer code

Data collection	All MS/MS data were collected using Mascot (Matrix Science, London UK, version 2.6.0), UniProt mouse database and the Scaffold program (Version 4.5.4, Proteome Software Inc, Portland, OR). Gene accession IDs of MS dataset protein/peptide annotations were obtained using UniProt (https://www.uniprot.org/uploadlists/). Mouse-human ortholog lists were collected using MGI (http://www.informatics.jax.org/), NCBI (https://www.ncbi.nlm.nih.gov/homologene) and Ensembl (biomaRt_2.42.1 and homologene_1.4.68 in R version 3.6.2; https://www.R-project.org/), which was aided by using R (details of the data collection steps and Rscript can be found in GitHub, https://github.com/CTR-BFX/2020-Napso_Sferruzi-Perri). Gene ontology analyses of lists of secreted placental proteins were collected using STRING and Panther tools (https://string-db.org/) and gene enrichment analyses were conducted using TissueEnrich (https://tissueenrich.gdcb.iastate.edu/). Lists of secreted proteins were obtained using SignalP (Signal Peptide Prediction, version 4.1). Data on transcription factor (TF) binding motifs was collected using EPD (Eukaryotic Promoter Database - https://epd.vital-it.ch/index.php), AME (Analysis of Motif Enrichment v4.12.0 - http://meme-suite.org/tools/ame) and Ingenuity Pathway Analysis (IPA, Qiagen). Heat map of MS peak areas of each peptide (normalized to the internal standard – bovine insulin) detected in mouse plasma was generated using Heatmapper (http://www.heatmapper.ca/expression/) using Average Linkage as clustering method and Spearman Rank Correlation as distance measurement method, applied to both columns and rows.
Data analysis	In addition to details provided in data collection section, statistical analyses were performed using GraphPad Prism version 7.00 (GraphPad Software). All data analysis steps can be found in GitHub (https://github.com/CTR-BFX/2020-Napso_Sferruzi-Perri).

For manuscripts utilizing custom algorithms or software that are central to the research but not yet described in published literature, software must be made available to editors and reviewers. We strongly encourage code deposition in a community repository (e.g. GitHub). See the Nature Research guidelines for submitting code & software for further information.

Data

Policy information about availability of data

All manuscripts must include a data availability statement. This statement should provide the following information, where applicable:

- Accession codes, unique identifiers, or web links for publicly available datasets
- A list of figures that have associated raw data
- A description of any restrictions on data availability

All source data underlying the graphs and tables are available in Supplementary Data files and/or can be found in GitHub (https://github.com/CTR-BFX/2020-Napso_Sferruzi-Perri). The mass spectrometry proteomics data have also been deposited to the ProteomeXchange Consortium via the PRIDE partner repository with the dataset identifier PXD025006 and 10.6019/PXD025006. All other data (if any) are available upon reasonable request.

Field-specific reporting

Please select the one below that is the best fit for your research. If you are not sure, read the appropriate sections before making your selection.

☒ Life sciences ☐ Behavioural & social sciences ☐ Ecological, evolutionary & environmental sciences

For a reference copy of the document with all sections, see nature.com/documents/nr-reporting-summary-flat.pdf

Life sciences study design

All studies must disclose on these points even when the disclosure is negative.

Sample size	We did not use statistical methods to calculate sample sizes, because the magnitudes of the effect sizes were not previously known. However, our sample sizes are similar to those reported in related publications in the field.
Data exclusions	No data were excluded.
Replication	We replicated all experiments at least five times (our sample sizes are 5 or more).
Randomization	Randomisation was not relevant to this study for either the secretome data or the analysis of placental proteins in human pregnancy samples
Blinding	Blinding was not relevant to to generating the secretome data as we did not have different groups to compare. Blinding was not possible for the proof of concept human study, as samples were retrospectively selected for this study. The purpose of the study was to develop a methodology and provide protein/gene lists for future investigation.

Reporting for specific materials, systems and methods

We require information from authors about some types of materials, experimental systems and methods used in many studies. Here, indicate whether each material, system or method listed is relevant to your study. If you are not sure if a list item applies to your research, read the appropriate section before selecting a response.

Materials & experimental systems

n/a	Involved in the study	
☒	☐	Antibodies
☒	☐	Eukaryotic cell lines
☒	☐	Palaeontology and archaeology
☐	☒	Animals and other organisms
☐	☒	Human research participants
☐	☒	Clinical data
☒	☐	Dual use research of concern

Methods

n/a	Involved in the study	
☒	☐	ChIP-seq
☒	☐	Flow cytometry
☒	☐	MRI-based neuroimaging

Animals and other organisms

Policy information about studies involving animals; ARRIVE guidelines recommended for reporting animal research

Laboratory animals	All mice used (virgin females and stud males, wild type and transgenic) were on a C57BL/6 genetic background. Females were 8-12 weeks old at the point of blood collection and/or time-mating (with subsequent blood and placenta collection). Stud males were at least 12 weeks old.
Wild animals	NA
Field-collected samples	NA

Ethics oversight

Mouse studies were approved by the University of Cambridge Ethical Review Panel and performed in accordance with the UK Home Office regulations Animals (Scientific Procedures) Act 1986 (project licence 70-7645 and PC6CEFE59)

Note that full information on the approval of the study protocol must also be provided in the manuscript.

Human research participants

Policy information about studies involving human research participants

Population characteristics

Pregnant women were retrospectively selected for this study from the Ophelia study (REC number 18/LO/0477 approved 5/4/2018). These pregnant women were matched for several factors, including body mass index, parity and gestational age. Clinical characteristics of the pregnant women are provided in table 1 of the manuscript.

Recruitment

Peripheral blood samples were retrospectively selected for this study from women recruited via the Ophelia study (REC number 18/LO/0477 approved 5th April 2018). Inclusion criteria included (1) singleton pregnancy, (2) no evidence of severe congenital anomaly and (3) a referral for an oral glucose tolerance test (OGTT) for clinical reasons, according to NICE guidelines (https://www.nice.org.uk/guidance/ng3). Exclusion criteria for this study were (1) multiple pregnancy (2) severe congenital anomaly on ultrasound, (3) severe anaemia on previous blood tests, (4) previous diagnosis of diabetes outside of pregnancy and (5) medications at the time of the OGTT, which may interfere with the results of the OGTT.

Ethics oversight

The methods were performed in accordance with relevant guidelines and regulations and approved by the Research Ethics Committee under agreement REC/LO/0477. All participants provided written informed consent prior to participation. Peripheral blood samples were retrospectively selected for this study from women recruited via the Ophelia study (REC number 18/LO/0477 approved 5/4/2018).

Note that full information on the approval of the study protocol must also be provided in the manuscript.

Clinical data

Policy information about clinical studies

All manuscripts should comply with the ICMJEguidelines for publication of clinical research and a completedCONSORT checklist must be included with all submissions.

Clinical trial registration

Ophelia study (Research Registry number 5528 and REC number 18/LO/0477 approved 5/4/2018)

Study protocol

Further details about the Ophelia study are provided in Meek, C. et al. Approaches to screening for hyperglycaemia in pregnant women during and after the Covid-19 pandemic. Diabetic Medicine, doi:10.17863/CAM.54385 (2020) and Meek, C. L. et al. Approaches to screening for hyperglycaemia in pregnant women during and after the COVID-19 pandemic. Diabet Med 38, e14380, doi:10.1111/dme.14380 (2021)

Data collection

Peripheral blood samples were retrospectively selected for this study from women recruited via the Ophelia study.

Outcomes

Primary outcome measure was a difference in the concentration or ratio of placental hormones in the circulation of pregnant women who went on to develop gestational diabetes compared to those who continued to have normal glucose tolerance during pregnancy.
